# An Unusual Case of Hepatic Tumor

**DOI:** 10.1155/1991/24946

**Published:** 1991

**Authors:** Antonio Frena, Alighieri Mazziotti, Gianfranco Zanetti, Giuseppe Gozzetti

**Affiliations:** 1 lstituto di Clinica Chirurgica II University of Bologna Bologna Italy; 2 lstituto di Anatomia Patologica University of Bologna Bologna Italy; 3 Istituto di Clinica Chirurgica, II Policlinico S. Orsola Via Massarenti, 9 Bologna 40138 Italy

## Abstract

A case is reported of a large hepatic tumor in a patient aged 71. Preoperative diagnostic techniques, including echography, CT and angiography, did not provide sufficient criteria for a precise diagnosis. The mass was removed with an extended right hepatectomy with no particular physiopathological consequences. Histological analysis revealed that this was a metastasis from a melonoma of the choroid, operated on 17 years previously.


*HPB Surgery*, 1991, Vol. 3, pp. 297–300
Reprints available directly from the publisher
Photocopying permitted by license only

© 1991 Harwood Academic Publishers GmbH
Printed in the United Kingdom

### AN UNUSUAL CASE OF HEPATIC TUMOR

ANTONIO FRENA, ALIGHIERI MAZZIOTTI, GIANFRANCO ZANETTI* and GIUSEPPE GOZZETTI

*Istituto di Clinica Chirurgica II, University of Bologna, Bologna, Italy*
**Istituto di Anatomia Patologica, University of Bologna, Bologna, Italy*

*(Received 1 June 1990, in final form 5 October 1990)*

A case is reported of a large hepatic tumor in a patient aged 71. Preoperative diagnostic techniques, including echography, CT and angiography, did not provide sufficient criteria for a precise diagnosis. The mass was removed with an extended right hepatectomy with no particular physiopathological consequences. Histological analysis revealed that this was a metastasis from a melonoma of the choroid, operated on 17 years previously.

KEY WORDS: Liver tumors, metastasis, eye tumors, choroidal melanoma

### INTRODUCTION

The possibility of developing hepatic tumors in the various stromal and vascular components of the liver, as well as in the hepatocytes and biliary ducts, accounts for the occasional observation of very rare forms of hepatic tumors. Very unusual forms of metastatic tumors may also be found since the liver is the preferential site for metastases of different types of tumors, frequently from outside the digestive tract, which, depending on their histogenesis, may reproduce polymorphic aspects which are sometimes difficult to interpret with preoperative diagnostic techniques.

### CASE REPORT

D.R., female, aged 71, underwent a medical check-up in November 1988 during which a palpable mass was found in the right hypocondrium. Her history is as follows: she had undergone hysterectomy for uterine leiomyofibromas (1964), right saphenectomy (1969), enucleation of the right eye (1972) for a neoplasia which she was unable to describe. Abdominal ultrasound, CT of the upper abdomen and hepatic arteriography revealed the presence of a large expansive cystic formation measuring 15 × 13 cm with solid areas within it, occupying the right liver lobe. The original hypothesis was that of a hepatic angioma with a vast central hemorrhagic area. Laparoscopy and a possible biopsy were refused by the patient. The liver function tests, the alphafetoprotein and CEA titre were always normal; the anti-hepatitis B antibodies were positive and the antigens negative. Due to the

Address for correspondence: Prof. Alighieri Mazziotti, Istituto di Clinica Chirurgica.II, Policlinico S. Orsola, Via Massarenti, 9, 40138 Bologna, Italy appearance of the lesion, which did not show clear neoplastic features, and given the age of the patient and the absence of symptoms, surgery was not suggested initially; an ultrasound follow-up was recommended to assess any changes in the volume and in the features of the lesion.

The subsequent, two-monthly echographic check-ups revealed a slight but constant growth in the size of the lesion, at a rate of approximately 1 cm every 2 months; however, at the check-up made in September 1989, the lesion measured approximately 19 cm at its maximum diameter, almost completely replacing the hepatic parenchyma of the right lobe and it had become symptomatic: the patient reported pain in the right hypocondrium especially after meals.

In November 1989 the patient was referred to us: ultrasound showed a further increase in the size of the mass which measured 22.5 × 17 cm; the echostructure was pleomorphic due to the presence of liquid and solid areas, the margins were clearly defined, there were no signs of infiltration of the hepatic parenchyma or of portal thrombosis. An abdominal angio-CT (Figure 1) was performed which revealed a lesion characterized by a solid peripheral component with scalloped edges and a mixed liquid-solid central component with areas separated by irregular septa.

The patient underwent surgery on 14.11.89: a long right subcostal incision revealed an enormous hepatic neoplasm, occupying almost the entire right lobe of the liver and mainly growing towards the posterior segments. Intraoperative echography revealed a mainly solid tumor, with large hemorrhagic areas inside it; there was no infiltration of the vascular peduncles nor of the remaining hepatic

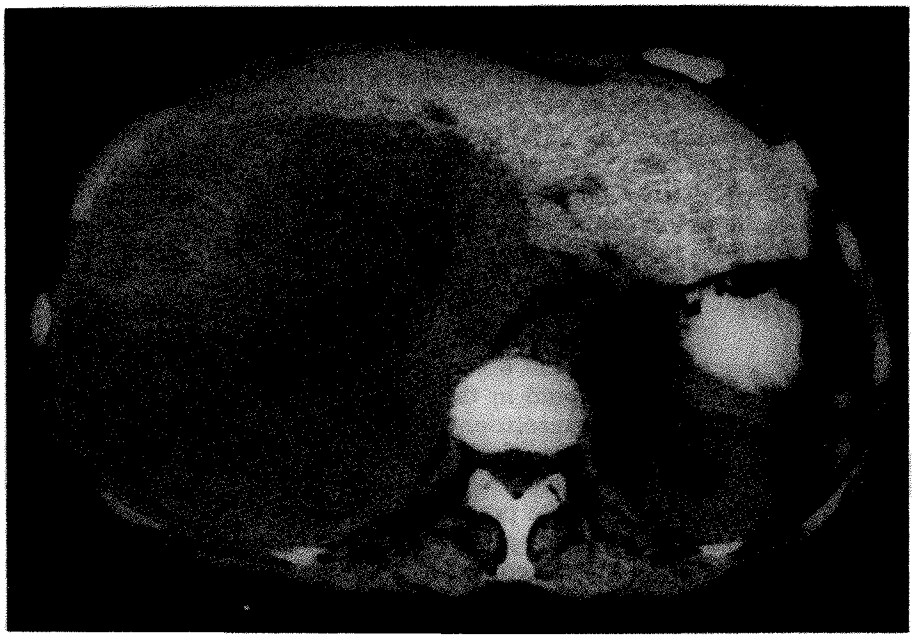

**Figure 1.** CT Scan with bolus contrast medium. Large intrahepatic mass with predominantly liquid central component and peripheral solid forms which have an uneven uptake of the contrast medium.

parenchyma where no pathological nodules were observed. An extended right hepatectomy was performed. The portal pedicle was clamped at the hilum (duration: 20 min.). The operation was performed without complications and lasted 240 min.; 2000 ml of blood and 1500 ml of plasma was transfused during the operation. The post-operative course was remarkably good. The patient was discharged on the tenth day after the operation.

Histopathology clearly revealed a fascicular pattern consisting of cohesive spindle cells. The mitotic rate was low. The positive argentaffin reaction of the pigmented cells and the evidence of melanosomes at an ultrastructural level confirmed the melanocytic origin of the tumor. The final histopathological diagnosis was of a spindle cell epitheloid tumor of melanocytic origin: metastases of a choroidal melanoma.

The hepatic echographic check-up made 12 months after the operation and chest X-rays showed no sign of new metastases.

### DISCUSSION

The frequency of eye tumors varies from 0.15% to 0.8% of all malignant tumors in various countries [1]; in Caucasians uveal melanoma accounts for 75–90% of eye tumors (with the exception of eyelid tumors)[2], whereas it does not represent more than 11% of melanoma in general [2]. As regards uveal localization, onset is most frequent in the choroid (93%), while melanoma of the iris (3%) and the ciliary body (4%) are much rarer. Surgery is the treatment of choice in these tumors and consists of enucleation of the eye: however half of the patients develop metastases in time, leading to death [2]. The time lapse between enucleation and the appearance of metastases varies a great deal and may be up to 30 years: in this case there was a time interval of 16 years between eye surgery and the finding of the hepatic mass; 99% of uveal melanoma metastases involve the liver [3], unlike cutaneous melanomas where secondary hepatic involvement occurs in 17% of cases. Very large retrospective studies [2] emphasize the extremely unfavourable prognostic significance of the metastases, even when they appear many years after enucleation of the primary tumor. In fact, in approximately 90% of the cases death of the patient occurs within one year of the appearance of clinical signs of secondary tumors. It is also extremely interesting that 43.2% of patients present with only hepatic metastases[4]. Sex and age of onset appear to have a certain importance for prognostic purposes: female sex and onset before the age of 60 have a more favourable prognosis: 35% mortality from the disease as compared to 50% for male patients or tumors with a later onset [5,6]. Prognosis is mainly influenced by primary tumor grading, according to Callender's original classification [7,8] or the more recent one by McLean [9]. The size of the ocular tumor is very important for the prediction of future metastases: small melanomas have a mortality rate for metastases over time equal to 15% whereas with larger ones the mortality is 45–50% [5,10].

The extreme rareness of these secondary hepatic tumors, especially in southern Europe, justifies the fact that this is the first hepatic resection for metastases from choroid melanosarcoma in our experience, which counts more than 80 resections for secondary tumors of the liver (71.4% of colo-rectal origin; 4.2% for digestive-endocrine tumors; 21.4% for neoplasms outside the digestive system) [11].

### *References*

1. Doll, R., Muir, C. and Waterhouse, J. (1970) Cancer incidence in five continents. International Union Against Cancer. Berlin: Springer Verlag
2. Raivo, I. (1977) Uveal melanoma in Finland. An epidemiological, clinical, histological and prognostic study. *Acta Ophtalmologica*, Suppl. **133**, 5–64
3. Pickren, J.V., Tsukada, Y. and Lane, W.W. (1982) Liver metastasis: Analysis of Autopsy Data. In *Liver metastasis*. Boston: Weiss L. & Gilbert H.A., Ed
4. Noor Sumba, M.S., Rahi, A.H.S. and Morgan G. (1980) Tumors of the Anterior Uvea. *Archives of Ophtalmology*, **98**, 82–85
5. McLean, M.J.W., Foster, W.D. and Zimmermann, L.E. (1977) Prognostic Factors in Small Malignant Melanomas of Choroid and Ciliary Body. *Archives of Ophtalmology*, **95**, 48–58
6. Shammas, H.F. and Blodi, F.C. (1977) Prognostic Factors in Choroidal and Ciliary Body Melanomas. *Archives of Ophtalmology*, **95**, 63–69
7. Callender, G.R. (1931) Malignant Melanotic Tumors of the eye: a study of histologic types in 111 cases. *Trans American Academy of Ophtalmology and Otolaryngology*, **36**, 131–140
8. Gamel, J.W., and McLean, M.J.W. (1977) Quantitative Melanoma Cells. *Archives of Ophtalmology*, **95**, 686–691
9. McLean, I., Zimmermann, L.E., and Evans, E. (1978) Reappraisal of Callender's spindle A-type of malignant melanoma of choroid and ciliary body. *American Journal of Ophtalmology*, **86**, 557–564
10. Apple, D.J. and Blodi, F.C. (1980) Advances regarding the Pathogenesis and Treatment of Ocular Tumors. *International Ophtalmological Clinic*, **20**, 33–40
11. Gozzetti, G. and Mazziotti, A. (1989) Expectation and possibilities of liver resection in the management of secondary liver tumors. In *Hepato-Biliary and Pancreatic malignancies*, Edited by Lygidakis & Tytgat, pp. 183–190. Amsterdam: Thieme Verlag.

*(Accepted by S. Bengmark 5 October 1990)*

